# Perspectives on Acceptance and Use of a Mobile Health Intervention for the Prevention of Atherosclerotic Cardiovascular Disease in Singapore: Mixed-Methods Study

**DOI:** 10.2196/11108

**Published:** 2019-03-14

**Authors:** Victoria Haldane, Yao Guo Tan, Krichelle Wei Qi Teo, Joel Jun Kai Koh, Aastha Srivastava, Rui Xiang Cheng, Yi Cheng Yap, Pei-Shi Ong, Rob M van Dam, Jie Min Foo, Falk Müller-Riemenschneider, Gerald Choon-Huat Koh, Pablo Perel, Helena Legido-Quigley

**Affiliations:** 1 Institute of Health Policy, Management & Evaluation University of Toronto Toronto, ON Canada; 2 Department of Pharmacy National University of Singapore Singapore Singapore; 3 London School of Hygiene and Tropical Medicine London United Kingdom; 4 World Heart Federation Geneva Switzerland

**Keywords:** atherosclerosis, mHealth, eHealth, patient-centered care, patient acceptance of health care, medication adherence

## Abstract

**Background:**

Cardiovascular disease, including atherosclerotic cardiovascular disease (ASCVD), is a growing public health threat globally and many individuals remain undiagnosed, untreated, and uncontrolled. Simultaneously, mobile health (mHealth) interventions using short messaging service (SMS) have gained popularity globally. There is an opportunity for innovative approaches such as mHealth to encourage and enable adherence to medications for ASCVD and its risk factors.

**Objective:**

This study aimed to understand mobile technology acceptance, use, and facilitating conditions among the study population ahead of the design of an mHealth intervention.

**Methods:**

Using data from a mixed-methods study conducted in Singapore, we conducted a cross-sectional survey with 100 participants and in-depth, semistructured interviews with 20 patients. All participants were over the age of 40 years with ASCVD or its risk factors. Interviews were conducted in English and Mandarin and if needed translated to English. Nvivo 11 (QSR International) was used for analyses.

**Results:**

Participants reported their perspectives on technology use and preferences, including low or sporadic mobile phone use and usability concerns including small screen and text size, among others; the benefit of previous mHealth use in creating a favorable opinion of SMS for health information; trust in both the source of mHealth SMS, as well as in treatment; the formation of habits; and fear of sequelae or death for facilitating intention to use an mHealth intervention and adhere to medication. We also highlighted a case that underscored the importance of the period after diagnosis in habit forming as an opportunity for an mHealth intervention.

**Conclusions:**

We explored both technology- and adherence-related factors that influence a patient’s intention to use an mHealth intervention for adherence to ASCVD medication in Singapore. We highlighted the importance of identifying the right opportunity to engage with patients and promote an mHealth intervention for adherence, such as immediately following diagnosis when patients are establishing medication-taking habits.

## Introduction

### Background

Mobile health (mHealth) interventions using short messaging service (SMS) have gained popularity as the number of people using mobile technology increases globally [1]. Singapore has one of the highest mobile phone penetration and usage rates in the world, thus providing an interesting case study for such interventions [2]. SMS-based interventions have shown promise because of affordability and wide outreach and have been applied to many aspects of health including health promotion and to enable medication adherence [3-5]. Although mHealth has provided many opportunities to reach patients, especially vulnerable groups, it is important that contextually appropriate patient preferences, usability, and acceptance of technology are considered when undertaking these interventions.

Patient preferences and acceptance are of particular importance when considering mHealth interventions for chronic conditions such as cardiovascular disease (CVD). CVD, including atherosclerotic cardiovascular disease (ASCVD), is a growing public health threat globally, with mortality rates estimated to reach 23.3 million by 2030 [6]. Importantly, many individuals with risk factors for ASCVD remain undiagnosed, untreated, and uncontrolled [7,8]. This points to the need for greater attention to factors impacting adherence in these patients and innovative approaches, such as mHealth, to encourage and enable adherence to medications for ASCVD and its risk factors in this patient population.

A systematic review demonstrated evidence of the feasibility of mHealth for adherence to medications for CVD. All 10 completed trials included in the review showed improved medication adherence for patients with CVD; all studies also reported positive responses to mHealth use from patients, however, the authors highlighted the paucity of data on studies including user input on the design of the intervention [9]. Qualitative studies have also shown the potential of mHealth to provide education, optimize resources, and improve use of health care for CVD management [10]. Yet, there is a need for more contextual evidence on patient acceptance and use of mHealth interventions for adherence, specifically exploring the unique factors that influence the use and acceptance of mHealth adherence support for medications to treat chronic conditions. For example, elderly patients managing chronic conditions may face unique usability concerns including functional difficulties using the device, limitations in vision and hearing, as well as the need for appropriate message content [11]. To explore the needs of those with or at risk for ASCVD in Singapore, our study population comprised patients over the age of 40 years identified as having ASCVD, or risk factors for ASCVD including hypertension or hyperlipidemia.

This study is the development phase of a proposed mHealth intervention, the txt2heart trial, to support patient adherence to medications for ASCVD. The txt2heart trial is an international collaboration evaluating the efficacy and safety of SMS on clinical outcomes and adherence in different countries including Colombia, Ghana, India, and Singapore. This study sought to explore mobile technology acceptance, use, and facilitating conditions, as well as adherence factors, among the Singapore study population ahead of the design of the mHealth intervention.

### The Study Setting: Singapore

To better contextualize our findings, it is necessary to consider larger health system factors relevant to Singapore, primarily the ubiquity of affordable and accessible health care. Singaporeans have access to largely subsidized care offered in polyclinics (government subsidized general practice clinics), private general practitioners, and tertiary care facilities for primary care and chronic condition management. Furthermore, within the primary care setting, doctors regularly prescribe months’ worth of medications, thus enabling ease of access to medications. The government also provides subsidies and various schemes for the management of chronic conditions; the majority of our participants reported availing these schemes to pay for their health care and most reported being able to afford and access their CVD medications.

### Theoretical Framework

**Figure 1:**
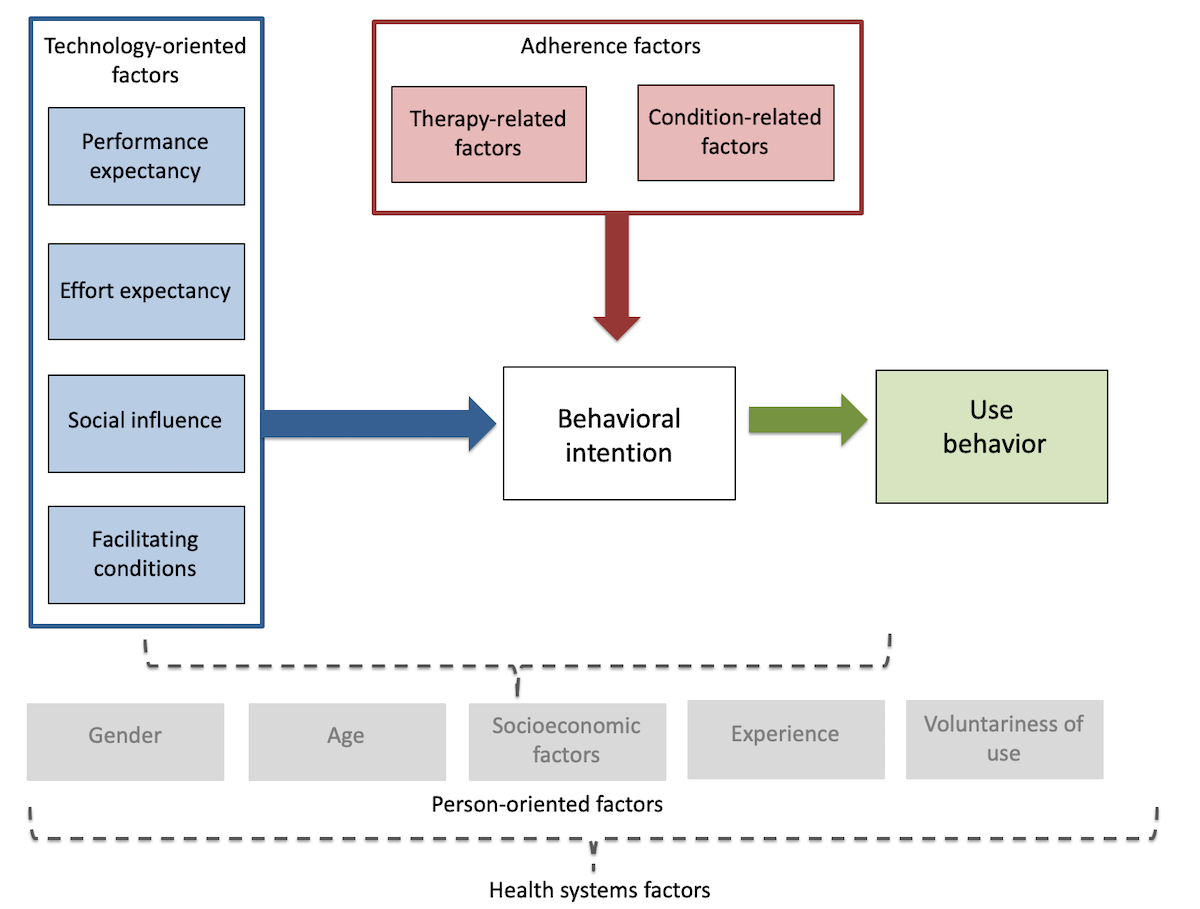
Modified unified theory of acceptance and use of technology model for adherence factors adapted from Venkatesh (2003) and World Health Organization (2003).

Using a deductive approach, we used the unified theory of acceptance and use of technology (UTAUT) model to analyze our qualitative findings. This was then complemented by emergent themes on medication adherence factors, which prompted us to apply the World Health Organization (WHO) adherence model to our data [12]. We then applied the modified and condensed UTAUT model to understand technology acceptance and use among participants with ASCVD and ASCVD risk factors (Figure 1). The UTAUT has been widely used to understand information technology (IT) adoption in general and has also been applied to health IT [13-17].

The UTAUT model employs 4 constructs through which to explore user factors; these being (1) performance expectancy, (2) effort expectancy, (3) social influence, and (4) facilitating conditions. Performance expectancy includes the perceived usefulness and personal outcome expectations associated with technology use. Effort expectancy is the perceived ease of use and complexity of the technology. Social influence includes subjective norms and technology use within a user’s social milieu, whereas facilitating conditions include perceived behavioral control and wider contextual circumstances that support the use of technology. We have labeled these as technology-oriented factors as they explore the users’ experience and preferences as oriented to their use and acceptance of technology.

Underpinning these constructs and impacting them in multiple complex ways are the influence of person-oriented factors including gender, age, socioeconomic factors, experience, and voluntariness of technology use. The confluence of these factors ultimately impacts one’s behavioral intention and use behavior as well as the actual use of the mobile technology or intervention.

An important modification necessary to understand use and acceptance of an mHealth intervention for adherence is the adherence factor, which may contribute to the behavioral intention and use of the intervention [18]. For our purposes, we have adapted the WHO framework to focus on the condition-related and therapy-related factors impacting adherence. Condition-related factors are the illness-related demands faced by the patient, which ultimately impact a patient’s risk perception, treatment beliefs, and the priority they place on adherence [18]. Therapy-related factors include side effects, treatment duration, treatment failures, and experience of side effects [18]. Although an intervention may meet the technology-oriented needs of a patient, the lived experience of their condition(s) and treatments could present barriers to their behavioral intention, both to adhere to treatment as well as to use an mHealth intervention to support treatment adherence.

In this study, we explore in detail the technology-oriented aspects of UTAUT as well as therapy- and condition-related adherence factors acting upon a patient’s behavioral intention. Thus, we use the modified and condensed UTAUT model and key elements of the WHO framework as a way to better understand the factors impacting elderly Singaporeans’ acceptance and use of mobile technology for an intervention to support adherence to ASCVD medication.

## Methods

### Sampling (Survey)

This cross-sectional component utilized data from a brief locally adapted survey created for this study, which sought to explore patient technology use. The survey used purposive sampling of an existing patient pool from the Singapore Population Health Study—Community Health Study to recruit those aged 40 years and above with established ASCVD or risk factors who fulfilled inclusion criteria (Textbox 1).

**Study inclusion and exclusion criteria.**

Inclusion criteria:

- Patients with a history of ASCVD: coronary artery disease, ischemic stroke, peripheral artery disease, and atherosclerotic aortic disease; or
- At least one risk factor such as hypertension or hyperlipidemia in whom antiplatelet, antihypertensives, and/or statins are recommended.

Exclusion criteria:

- Participants unable to participate in a verbal interview;
- Participants who did not speak Mandarin, English, or Malay languages.

Both the survey and interview guide were developed as part of the larger txt2heart collaboration, which we then adapted to the Singapore context and covered topics including patients’ sociodemographic characteristics, health care access, medication adherence, mobile phone technology usage (ownership, access, and utilization), and interest in mHealth (Multimedia Appendix 1).

### Sampling (Qualitative)

The study took place in Singapore. We used purposive sampling from an existing patient list to recruit those aged 40 years and above with established ASCVD or risk factors for ASCVD (for complete inclusion and exclusion criteria, see Textbox 1).

### Survey, Interviews, and Interview Guide

Trained research staff from the National University of Singapore (NUS) conducted the interviews or semistructured in-depth interviews in the participant’s preferred language by staff fluent in that language. Interviewer training included description of research protocol, qualitative methods, and research ethics in practice.

### Participants

The qualitative component involved semistructured in-depth interviews with 20 participants. Out of the 20 participants interviewed, 19 agreed to audio recording and 1 participant declined. For the latter, detailed field notes and an extensive memo were taken for inclusion in data analysis.

A total of 100 patients met the inclusion criteria for the quantitative survey and were recruited over the telephone. In total, 60 in-person and 40 telephone surveys were completed following informed consent from patients. A total of 100 surveys, inclusive of 1 incomplete survey on mobile phone technology usage section, assessed patients’ sociodemographic characteristics, pattern of medication adherence, mobile phone technology usage (ownership, access, and utilization), and interest in mHealth.

### Ethical Approval

Ethical approval for the study was obtained from the NUS Institutional Review Board. Informed consent for participation and recording was obtained before the interview started using a participant information sheet and consent form. Participants could refuse to answer any of the questions and/or discontinue their participation in the research at any time.

### Statistical Analysis

Statistical analyses were performed using IBM SPSS version 24.0 (IBM Inc). Frequencies (n) and percentages (%) were used to summarize sociodemographic characteristics, clinical characteristics, patterns of mobile phone technology usage, and interest in mHealth.

### Qualitative Analysis

A total of 2 research team members coded interviews using Nvivo 11 (QSR International) software applying interpretive and inductive approaches, thematic analysis, and techniques from the constant comparative method, where a line-by-line analysis of early interviews was used on subsequent interviews to test preliminary assumptions [19]. Interviews were recorded and transcribed in full. Reviewers agreed on identified codes and themes. To maintain confidentiality, all names reported were pseudonyms and identifying data were excluded.

## Results

### Participant Characteristics

The mean age of the 100 participants in the analysis sample was 65.3 (SD 9.6) years (Table 1). The largest proportion of participants, 55% (55/100), encompassed those aged greater than or equal to 65 years. The majority of patients (70/100, 70%) were male and 66% (66/100) were of Chinese ethnicity, with women and Malay, Indian, and other ethnicities representing a smaller proportion of the sample. Moreover, the majority of patients, 72% (72/100), reported having hyperlipidemia and 68% (68/100) reported having hypertension.

We conducted 20 in-depth interviews with participants who met the inclusion criteria. The detailed characteristics are presented in Table 2. Participants were largely of Chinese ethnicity (15/20, 75%) with fewer participants from Indian (4/100, 20%) and Malay (1/20, 5%) ethnic groups. Participants were mostly male (12/20, 60%) with 8 (8/20, 40%) female participants. The average age of participants was 72.5 years. The majority of participants (19/20, 95%) reported a hypertension diagnosis, followed by hyperlipidemia (16/20, 80%) and myocardial infarction/acute coronary syndrome (8/20, 40%) and stroke/transient ischemic attack (5/20, 25%).

**Table 1:** Quantitative participant characteristics table (n=100).

Sociodemographic characteristics		Total (n)
**Ethnicity**		
	Chinese	66
	Malay	19
	Indian	14
	Other	1
**Gender**		
	Male	70
	Female	30
**Age (years)**		
	<65 years old	45
	≥65 years old	55
**Cardiovascular conditions**		
	Hyperlipidemia	72
	Hypertension	68
	Myocardial infarction or acute coronary syndrome	41
	Stroke or transient ischemic attack	14

**Table 2:** Qualitative participant characteristics table (n=20).

Sociodemographic characteristics		Total (n)
**Ethnicity**		
	Chinese	15
	Indian	4
	Malay	1
**Gender**		
	Male	12
	Female	8
**Age (years)**		
	61-70	7
	71-80	8
	81-90	4
	Missing	1
**Cardiovascular conditions**		
	Hypertension	19
	Hyperlipidemia	16
	Myocardial infarction/acute coronary syndrome	8
	Stroke/Transient ischemic attack	5

### Quantitative Survey Results

**Figure 2:**
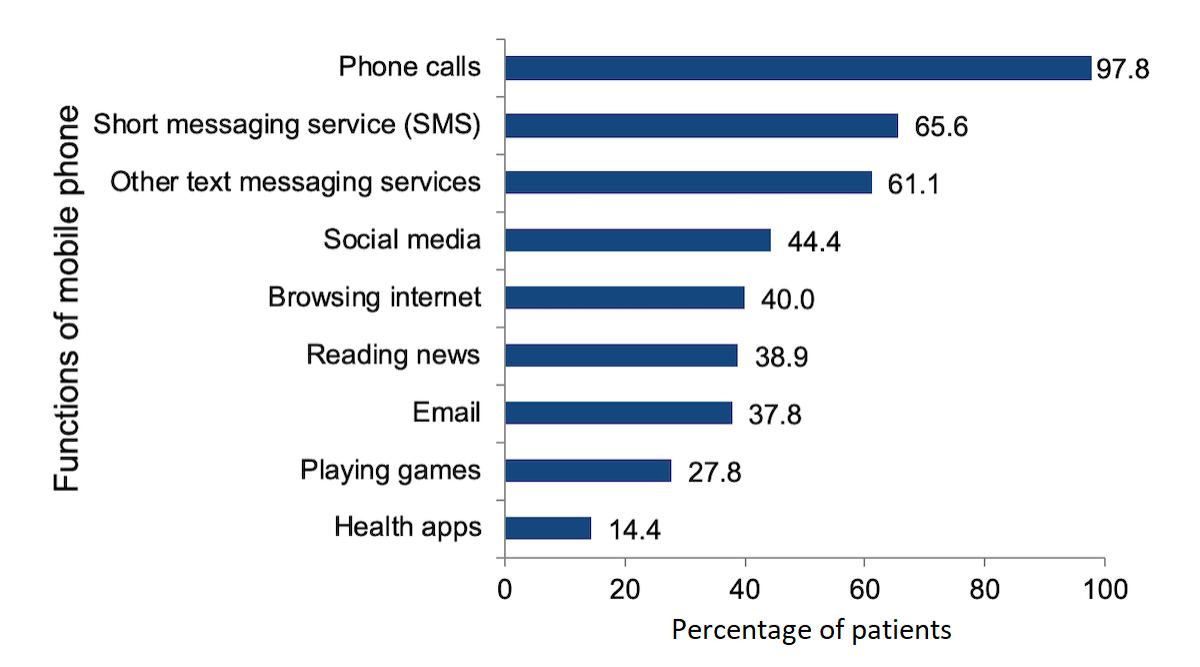
Phone usage activities amongst mobile phone owners.

A quantitative survey of 100 patients meeting the same criteria outlined above provided our study with descriptive data on our target population. Of the 99 participants who fully completed the survey, 90% (90/99) owned a mobile phone. Of those mobile phone owners, 77% (70/90) reported accessing their mobile phones in general at least once a day, and the same proportion of patients (70/90, 77%) reported using SMS at least once a day. In general, participants predominantly used their phones for phone calls (87/90, 97%), SMS (60/90, 65%), and other text messaging services such as Whatsapp (54/90, 61%; Figure 2).

**Figure 3:**
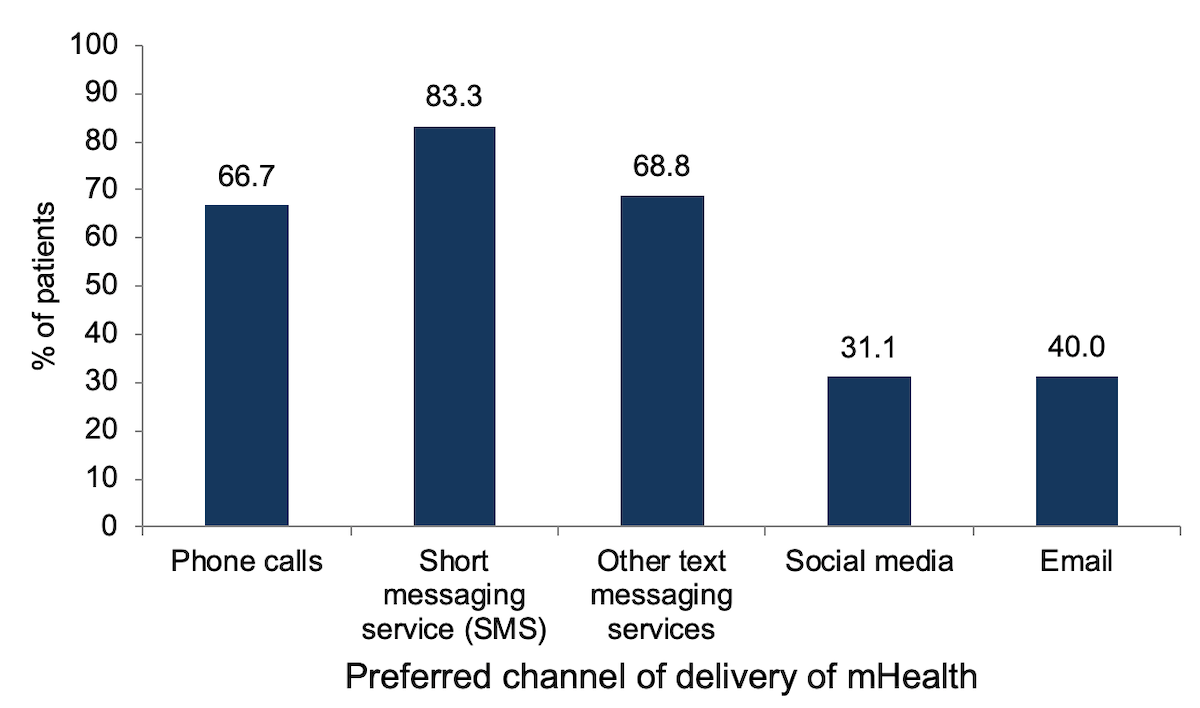
Preferred channel of delivery for mobile health intervention. mHealth: mobile health.

Of those who owned a mobile phone, 53.3% (48/90) indicated their interest in receiving medication information through their mobile phones.

Among those interested in mHealth services, SMS was the preferred mode of delivery of information on medication for 40 out of 48 participants (83%), followed by other text messaging services (33/48, 68%), phone calls (32/48, 66%), social media such as Facebook and email (15/48, 31%; Figure 3).

### Qualitative Interview Findings

We reported our findings classified into themes based on our modified UTAUT model. In terms of technology-oriented factors, the first theme explored perceived usefulness of the mHealth intervention and personal outcome expectations. The second theme explored effort expectancy, including perceived ease of use, as well as other usability issues experienced by our elderly population. Next, we explored the social influences shaping technology use including the role of family and friends, as well as the impact of social isolation. Then we looked at facilitating conditions for technology use including trust and previous mHealth use. We next explored adherence factors influencing behavioral intention, including therapy-related factors such as regime complexity, therapy duration, inconvenience, and adverse effects. Finally, we looked at condition-related factors including symptoms and effects of condition on the functional and mental state. See Multimedia Appendix 2 for key themes and examples of evidence.

### Perceived Usefulness

Participants reported on their perception of the usefulness of having a reminder system to take their medications. Most participants reported that they saw no need for such a system, as they did not have difficulties remembering. As Yong Liat reported:

I can’t forget. Every time I put it on top, and I write down there. So I know. People don’t need to tell me. I know myself.

IDI006_M_81-85_Chinese

However, some participants did agree that hypothetically having reminders may be good for others who forget to take their medications. As Kim Huat explained:

They should see the message and it should help. After all the reminder for them to take the medication is actually beneficial for them, and they should heed the reminders.

IDI009_M_71-75_Chinese

Some participants also voiced that low phone usage may inhibit the usefulness of an SMS intervention. As Irene described:

I don’t look at my phone...when the phone beeps with an SMS I’ll think of checking it later, but I’ll forget. Do you see how many unread and unanswered SMSes and WhatsApp message I have?

IDI004_F_66-70_Chinese

### Personal Outcomes Expectations

The majority of participants explained that they personally felt an SMS intervention would not be useful or provide a useful outcome. This is partly because of some participants having low reliance on their phones, meaning the vehicle for delivery may not be aligned with the population’s mobile uses. Furthermore, some participants expressed that the intervention does not address what they perceive to be the root causes of nonadherence, which they feel to be beyond the reach of mHealth. For example, an SMS intervention would not help those who intentionally do not take their medication. As Ah Siew explained:

If they want to take medication, they will. Some people they’re not taking it on purpose. I don’t take some of my medication too. We take only the important medicine and don’t take those that we feel aren’t important.

IDI020_F_71-75_Chinese

Another participant highlighted that regardless of the proposed intervention, a key factor in adherence is patient agency and responsibility in placing importance on their health. Yan Ting explained:

I think whatever method you want to use, it's nothing compare to convincing that patient that it is in their own interest and it is important for them to take the medicine regularly according to the schedule... I think if they are serious looking after themselves, it becomes a habit already.

IDI018_M_76-80_Chinese

However, some participants reported on factors that may be modifiable by an mHealth intervention. Irene summed up her perspective of the main factors for nonadherence, forgetfulness, dependency on others, and being busy:

Interviewer:
*How do you think we can use mobile phones to remind people to take their medication?*

Participant:
*When people forget to take their medications it’s the following three reasons. First, they’re forgetful, like me. I tell myself it’s time to take my medication and walk to the kitchen. When I’m in the kitchen, I can’t remember why I came into the kitchen. Second, they’re dependent. They wait for people to tell them. Third, they are too busy. Just these three types of people.* [IDI004_F_66-70_Chinese]

### Effort Expectancy

As mHealth interventions predicate on patients being able to interact with SMS, it is important to understand their perceptions of basic mobile technologies as well as their perceptions on their ability to learn how to use such technologies.

#### Perceived Ease of Use

Some participants described difficulty using a mobile phone, thus preventing them from being interested in an mHealth intervention. However, others were keen to keep up with technology and expressed initiative and interest in learning about their phones. Safiah explained how she previously did not know how to use some features of her phone, but now is able to:

Previously I SMS right-- previously aunty don’t know how to SMS...So now everything I know. You live in the world of today...Every now and then, you must go out and learn everything. What the youngsters can do, the old also can do.

IDI012_F_71-75_Malay

Among those who are comfortable using technology, a few reported that SMS is a good and convenient way to convey health information:

It’s also good via SMS or WhatsApp since it’s more convenient and the information is in the phone. Sometimes we might not have time to answer a call.

IDI009_M_76-80_Chinese

#### Usability Issues

Many participants, however, reported on functional issues preventing them from comfortably using a mobile phone including small screens or font sizes that are barriers to reading SMS messages. As Cheng Han explained:

Our eyesight is also failing. Sometimes we see an“8” as an “S.”It also makes it difficult to read our SMS. Our senses all deteriorate after 70 years old.

IDI014_M_71-75_Chinese

Another usability concern raised by many participants was that of the language of intervention. Most participants agreed the SMS reminders should be sent in local languages. As Kim Huat reported:

It would help if it’s in Chinese, Malay, Tamil. Those of us who are older don’t understand English, and thus the content in the SMS.

IDI009_M_76-80_Chinese

While Sow Tin explained that some older people had lower literacy and thus require simplified SMS content:

Firstly, I didn’t really go to school. So I can only read really simple English and Chinese. I can speak better, but not write...I will just reply “Ok.” If I don’t know how to text back my response, I will send them a voice message. It’s easier like that.

IDI004_F_66-10_Chinese

### Social Influence

Another important component of technology uptake and use is that of social influence. Those with stronger social networks who use and look favorably on technology may use technology to connect and stay in touch, and these networks may in turn enable them to adopt technology.

### Family and Friends’ Technology Use

Some participants reported that the lack of a social support system is a reason for not being interested in using a mobile phone, and others reported using the phone less as their friends had passed away. As Wen Cheng explained:

I don’t have many friends. I don’t need to talk a lot now, or I just use the home telephone.

IDI001_M_71-75_Chinese

Among those who had stronger social supports, many described that their friends were an important factor in their ability to learn and keep up with technology. Others described how technology had enabled them to keep in touch with friends from abroad and that these groups were helpful to them, thus giving them a favorable impression of mobile technology. One participant described how technology and social support played a role in adherence. As Kavita reported:

She (friend) every time calls me, you know, because she know I forget.“Hey, take medicine already?”She don’t know which medicine I take but she give me a reminder.

IDI013_F_61-65_Indian

### Facilitating Conditions

Our participants highlighted the importance of previous exposure to mHealth services and trust as facilitators to mHealth use.

#### Previous Mobile Health Use

A majority of participants reported positive experiences with previous mHealth apps, namely SMS appointment reminders. Participants felt these were useful and helped them to remember their appointments. Kim Huat explained:

They will send SMS to me, and they also give me a paper with the appointment date. They usually remind me via SMS a week before my appointment...It’s a good reminder so that you don’t forget.

IDI009_M_76-80_Chinese

One participant, Kamal, highlighted that patients may not be an appropriate audience to target the SMS reminders to and it would be more useful to engage primary caregivers in mHealth interventions:

Tell the person looking after the patient to remind them to take their medication...The person cannot remember...so if you tell the children or (caregiver) they will make sure they take the medication, as if the orders come from the doctor the caregiver will scared and make sure they take.

IDI010_M_71-75_Indian

#### Trust

An important facilitating factor reported by patients was that of trust. Some patients explained that they do not trust all the information they reiceve on the Web or shared through Whatsapp groups. As Kim Huat said:

There are a lot of fake information there (on the phone), not all are true.

IDI009_M_76-80_Chinese

Thus, highlighting the need for an mHealth intervention to come from a trusted and known source.

Kim Huat went on to explain how the underlying trust in treatment is important in an mHealth intervention for adherence to medications:

Interviewer:
*Who do you think this service will help?*

Participant:
*Those that believes that their medication is effective. After all if you face any issues while taking the medication, the doctor and change it for you.* [IDI009_M_76-80_Chinese]

This highlighted the need to not only understand user preferences and behaviors, but also to take into consideration the various adherence factors, which influence patient decision making and ability to adhere to medications.

### Adherence Factors

Adherence factors include therapy-related and condition-related factors. To design an appropriate mHealth intervention, it is important to understand how both the medication itself and the condition may limit or enable adherence to medication.

#### Therapy-Related Factors

##### Complexity of Medication Regime

Some patients reported adjusting their medication to cope with a complex regime and to facilitate their ability to adhere to their medications. As Sow Tin described:

The doctor did tell me to take the blue pills before food. I thought it was troublesome to split them, so I took all my pills before food. So I can remember whether I’ve taken my medication...The doctor did say I was cheating. But it’s okay. It’s more time efficient for me.

IDI004_F_66-70_Chinese

Nearly all patients described how using a pillbox and the ritual surrounding taking their medication had enabled them to form habits. Patients also reported that they did not feel the need to have an mHealth intervention to remind them as their current habits and pillbox use enabled them to ensure they had taken medications and prevented them from taking a double dose.

##### Duration of Therapy

The majority of patients also reported how the long duration of therapy for chronic conditions facilitated their ability to adhere to their medications. Thus, they felt that an mHealth intervention would not be of use, as they had already established habits over the course of managing their chronic conditions. As Karen reported:

I just follow...because I have been using it for long, it’s no problem to me...It’s all very systematic. No problem.

IDI003_F_NA_Chinese

##### Inconvenience With Lifestyle

Although most patients reported that they largely adhered to their medications and had habits to support their medication taking, some patients reported how lifestyle factors disrupted their routines on occasion. Largely, patients reported how going out of the house or travelling was disruptive. As Ah Siew described:

But if I go out, I won’t bring and eat it. It’s very troublesome to take it when I’m on the bus or (train). I’ll just miss taking it. For instance, I went to Ipoh Malaysia the other day, I brought my medicine but I didn’t take it for two days, as it’s too troublesome.

IDI020_F_71-75_Chinese

##### Adverse Effects

Some patients reported how adverse effects caused them to be nonadherent to their medications. One participant reported that while the doctor wanted to titrate the dose, he decided to stop entirely. As Yong Liat reported:

Just recently. Because I told the doctor, I feel giddy then doctor say maybe this one caused...he asked me to take half...so I stopped.

IDI006_M_81-85_Chinese

#### Condition-Related Factors

Patients reported few condition-related factors as impacting their ability to adhere to their medications for ASCVD and its risk factors. One patient reported being prescribed various doses of medication and not taking it unless his blood pressure was high. As Cheng Han described:

Yes. I may forget my blood pressure medications...For blood pressure, I usually don’t take the medications unless my blood pressure is high.

IDI014_M_71-75_Chinese

A few participants also reported how taking medications impacted their mental state, as they felt fearful of sequelae or death. For some participants, this caused them to titrate or change medications. As Sow Tin described:

You see, this cholesterol medication was given to me in March, and I still have so much leftover. If I show the doctor, he/she is going to scold me. So I won’t tell the doctor...My sister told me that if I take a lot of these cholesterol medication, it will affect my kidneys. So I’ve stopped taking that one and take this one instead. I’m not as afraid of kidney trouble with this one.

IDI004_F_66-70_Chinese

Whereas, others reported that fear of disability and death motivated them to continue their habits and be adherent to their medications. As Yu Yan explained:

I take them right after washing my face as I scared I forget...It’s okay to die, it’s not okay to be disabled. So I take my medications...Because I’m scared to die, it motivates me to eat my medication, without even any reminder.

IDI008_F_71-75_Chinese

### The Learning Curve and Mobile Technology

One participant highlighted in detail the learning curve after diagnosis and commencement of treatment as a potential opportunity for mHealth interventions for adherence to medications (Textbox 2).

This case explored how the uncertainty of a new diagnosis and discharge from hospital care is an opportunity for mHealth to provide adherence support to patients where they have a high intention to uptake adherence behaviors. Patients who have managed their chronic conditions for years may not be as receptive as they have established habits; however, newly diagnosed patients or newly discharged patients may be primed to receive support in their care.

**Case study on the learning curve.**

David, a 64-year-old male, had no history of heart disease before having a severe heart attack, which required the insertion of a left ventricular assist device. He was admitted to the intensive care unit for over a month before being discharged. While in hospital, David reported being on 14 different types of medication, which were reduced to 11 upon discharge after he reported adverse side effects. Presently, he is only on 2 medications. David explained the role of his care coordinators, who taught him about his medications before discharge. He also explained how they interceded on his behalf with the doctors to reduce the number of medications he was prescribed. Importantly, he described how they were available to call or SMS when he had questions. When asked about the use of an mHealth intervention to remind patients to take their medications, he explained: “It will be useful for patients who just came out of the ward. But not for long-term patients, because we know what to do.” [IDI007_M_61-65_Indian]; David reported that in the days following discharge, the care coordinators would check in daily and ask on his adherence and other required activities (dressing changes, etc); and now they check in every 2 weeks, and he is able to reach out to them should he require their assistance.

## Discussion

### Principal Findings

This mixed-methods study explored mobile technology acceptance, use, and facilitating conditions, as well as adherence factors among older Singaporeans ahead of an mHealth intervention to promote adherence to ASCVD medications.

Participants reported variable acceptance of mobile technology. Some participants were tech savvy and used mobile technology regularly and broadly to connect with their social networks, whereas others reported decreasing social connectedness as a reason for not using mobile technology. Participants widely reported usability concerns including reading difficulty because of screen and font size and hearing difficulties limiting their awareness of notifications. This is in line with other research on mobile technology use for health interventions with older participants [20]. These technology-oriented factors have an impact on user’s behavioral intention to use technology, as well as their intention or interest in using an mHealth intervention for adherence to medications. Indeed, among our study population, whose mean age was 72 years for the qualitative component, participants were largely disinterested in SMS reminders, often citing low mobile phone usage or usability concerns as a barrier to uptake.

Suboptimal adherence to medications for ASCVD and its risk factors is well documented [6,21-23]; however, most of our participants reported not needing reminders to take their medication as they have their own established habits and report themselves as being adherent. Yet, some participants reported self-titration and nonadherence when lifestyle factors interfered with their ability to have medications on hand. Thus, caution is warranted in these self-reports of adherence. These reports of adherence may impact patients’ behavioral intention to use an mHealth intervention, as many participants reported no interest in an mHealth intervention for adherence to medication, as they did not see personal benefit to such a service.

Despite these concerns, some participants did report that SMS was a good avenue through which to receive health information. This is in line with findings from other studies across health research where it has been shown that despite perceived usability issues or dissatisfaction with available options, patients believe in the capacity of mHealth interventions to facilitate better health outcomes [24-27]. In our study, a contributor to a favorable opinion of the proposed intervention was previous mHealth use. Participants who received appointment reminders from health care providers were more open to the idea of health content delivered through SMS, although they were ambivalent on the utility of medication reminders.

Another factor facilitating a positive perception of mHealth was that of trust; both trust in the SMS sender, and trust in the treatment plan. Participants described caution in believing Web-based sources for health information, reported receiving spam SMS messages, and underscored the importance of wanting a trusted source for any mHealth SMS. Other studies have shown that trust is an integral part of intent-to-use technology and predicates on the belief that the other party will not exploit the vulnerability of the user [28,29]. Participants also linked the success of any mHealth intervention to whether patients trusted their medications. There is ample evidence on the role of trust, both in treatment and in provider, in medication adherence [30-32], and these facets of trust in treatment are important in establishing the behavioral intention to use an mHealth intervention for adherence to medication.

Although technology-oriented factors directly influence a patient’s ability and motivation to use and follow an mHealth intervention, adherence factors also play a role in determining if a patient is able to translate the behavioral intention triggered by the SMS into a use behavior. The use of habits and reminders is well documented in facilitating adherence to medications [33-35]. Our study adds to this evidence showing that patients perceive they are able to cope with treatment complexity by establishing their own habits and rituals and that given the length of treatment required they become accustomed to taking their medications. Importantly, however, patients did report self-titration because of adverse effects, as well as lifestyle factors that disrupted their ability to adhere to medications. These are important factors that may not be modifiable by an mHealth intervention but warrant consideration.

The unique case of David highlights the importance of the learning curve wherein patients develop these habits and rituals for medication taking. After diagnosis, patients may require additional support and be open to receiving that support, as they begin to develop habits and adopt behaviors relating to medication taking and lifestyle adjustment. Although, as is the case with David, this support may taper off over time once adherence behaviors are established, or the perceived utility diminishes. It also presents the opportunity to reintroduce the intervention should new medications be introduced or the patient requests additional support. Although technology-oriented factors, adherence factors, and contextual factors may pose challenges to uptake of an mHealth intervention, this case highlights the importance of identifying opportunities where the barriers to mHealth uptake may be lower as patients are actively seeking support as they take ownership of their care.

### Strength and Limitations

A strength of our study is the use of in-depth interviews for the exploration of patients’ perspectives on both medication taking, as well as technology use and the opportunities and challenges for mHealth interventions. Furthermore, the inclusion of participants from multiple ethnic backgrounds and older participants adds to the diversity of experiences reported.

A limitation of this study is that it excluded individuals with disabilities, which prevented them from participating in verbal interviews. Also, our participants were all over 60 years of age, thus we are not capturing the perspectives of those middle-aged persons taking medications for ASCVD and its risk factors who may have differing adherence patterns or mobile technology use behaviors. The fact that Singapore has a high mobile phone penetration rate, a small geographical size, and is a 100% urbanized city-state may limit its generalizability to other countries with differences in such characteristics. Furthermore, contextually, health system factors in Singapore, including high accessibility and availability of care may account for some of the patient accounts of not needing reminders, as they are able to easily access follow-up care. Our study also did not include caregivers, who may be a more appropriate end user for the intervention, particularly among those older persons who rely on caregivers for their medication taking. Furthermore, we relied on self-reported adherence measures, thus some participants may have reported higher adherence to medication. A final limitation is that of desirability bias, whereby participants may be reporting more favorably on their experiences.

### Conclusions

Our study had identified several important technology-oriented and adherence-related factors from the patient perspective that warrant consideration in the design of an mHealth intervention to support adherence to medications for ASCVD and its risk factors in Singapore. We also highlighted the importance of finding the right opportunity to engage with patients and promote an mHealth intervention, such as immediately following diagnosis when patients are establishing medication-taking habits. As health care professionals increasingly leverage on innovative approaches such as mHealth to promote adherence to medications for chronic conditions, it will be important to better understand both the technology-related behaviors that impact a patient’s intention and ability to use an mHealth intervention, as well as therapy- and condition­-related factors that may enable or inhibit successful adoption of such an intervention.

## Appendix

Multimedia Appendix 1 Summary qualitative interview guide.

Multimedia Appendix 2 Key themes and examples of evidence.

## Abbreviations

ASCVD: atherosclerotic cardiovascular disease
CVD: cardiovascular disease
IT: information technology
mHealth: mobile health
NUS: National University of Singapore
SMS: short messaging service
UTAUT: unified theory of acceptance and use of technology
WHO: World Health Organization
