# Supplementary material for: Perspectives on Acceptance and Use of a Mobile Health Intervention for the Prevention of Atherosclerotic Cardiovascular Disease in Singapore: Mixed-Methods Study
Source: JMIR Mhealth Uhealth. 2019 Mar 14;7(3):e11108. doi: 10.2196/11108 (PMC6437612; doi:10.2196/11108)
Supplement: Multimedia Appendix 1 [file mhealth_v7i3e11108_app1.pdf]

Multimedia Appendix 1. Summary qualitative interview guide.

Introductory Questions:<br>• Are you currently employed?<br>• Take demographic information<br>• To start, could you tell us a bit about your family?<br>• Can you tell me a bit about your living situation? Do you live with anyone? Who?<br>• Are you currently employed?<br>• Does anyone help you with your daily tasks like chores or cooking?
Medical History and perception of condition<br>• Could you tell me about your health history (or medical conditions you have)? [*Follow their narrative to explore the patients' journey*]<br>• Can you tell me about how you were diagnosed with Cardiovascular/heart disease?<br>• Could you share with me the changes you had to make in daily life after you were diagnosed with CVD/heart disease?<br>• What do you think contributed to your CVD/heart disease?<br>• What did you know about CVD/heart disease before you were diagnosed?
Perception and taking medication<br>• Could you share with us all the medicine you currently take? *[Ask if it is possible for us to see the medication, and ask about when and how often they take it]*<br>• What instructions were you given on how to take the medication<br>• Do you feel like taking the medication helps to relief of your condition?<br>• Do you take your medication regularly?<br>• Do you experience any problems in taking the medication?

- Do you ever forget to take your medicine?
- Does anyone or anything remind you to take your medication?

Health System Perception

- Moving on, can you tell me about where you go to see the doctor for your CVD/heart disease?

- How often do you see your doctor?

- Overall, what is your relationship with your doctor like?
- Do you talk to any other health care workers for your CVD/heart disease? Nurses? Counsellors? Can you tell me about your relationship with them?
- Do you face any difficulties accessing health services?
- How do you pay for your medical expenses?
- Do you have any problems affording for your CVD/heart disease medication?

Support and information networks

- Where do you seek information about how to manage your CVD/heart disease?
- What kind of information?
- Do you use this information frequently?
- Do you feel like you have enough information to help you manage your CVD/heart disease?
- Does anyone help you manage your CVD/Heart Disease?

Use and appropriation of technology

- Do you own a mobile phone?
- How often do you use your mobile phone?
- What do you usually use your mobile phone for?

- Do you experience any difficulties using your mobile phone?
- Would you find it useful to receive information about different aspects of your treatment through your mobile phone?
- Would you find it useful to receive reminders to take your medication through your mobile phone?
- How often would you like to receive such reminders?
- Would you like the possibility of being able to reply to these messages, where healthcare professionals can respond to your queries?
