# Supplementary material for: Perspectives on Acceptance and Use of a Mobile Health Intervention for the Prevention of Atherosclerotic Cardiovascular Disease in Singapore: Mixed-Methods Study
Source: JMIR Mhealth Uhealth. 2019 Mar 14;7(3):e11108. doi: 10.2196/11108 (PMC6437612; doi:10.2196/11108)
Supplement: Multimedia Appendix 2 [file mhealth_v7i3e11108_app2.pdf]

Multimedia Appendix 2. Key themes and examples of evidence.

Modified UTAUT Constructs		Examples of Evidence
Technology-Oriented Factors		
Performance Expectancy	Perceived usefulness	*"For me, I can read the instructions and I know the medicine and dosage. So I don't need people to inform me doing this and that. I can just read from the bottle." IDI003_F_NA_Chinese*
	Personal outcome expectations	*""People won't bother. You ask me to do it, give it to me, you can't incentivize me to do it because it's a hassle." IDI015_M_61-65_Chinese*
Effort Expectancy	Perceived ease of use	*""I've heard that we need to chase up with technology. But do you think everyone went to school? If you teach me what this is, I'll forget what that was." IDI005_F_81-85_Chinese*
	Usability issues	*"Firstly, the numbers are very small, my fingers are very thick, so two numbers would be dialled at the same time." IDI002_M-81-85_Chinese*
Social Influence	Family/friends technology use	*"I can't be asking my children all the time, they will be annoyed. So I sit with my friends and they will slowly explain it and I will understand." IDI009_M_76-80_Chinese*
Facilitating Conditions	Previous mHealth use	*"I think one good thing is that it can remind me when to do the check-up and follow up. It's a*

		very good service…These reminders are very good." IDI003_F_NA_Chinese
	Trust	*"With apps, you don't know who's on the other side, a person with no name." IDI007_M_61-65_Indian*
Adherence Factors		
Therapy Related Factors	Complexity of medication regime	*"I told them best not to ask me to take 1.5 pills. It's hard to cut a pill…" IDI005_F_81-85_Chinese*
	Duration of therapy	*"No, cannot be [confusing to take]. 'Coz I'm already used to them. I've been taking them for 14 plus years." IDI010_M_71-75_Indian*
	Inconvenience with lifestyle	*"I tend to forget my medications in the morning on weekends. I take care of my grandchildren, I go to Woodlands where my daughter stays, and so I forget." IDI004_F_66-70_Chinese*
	Adverse effects	*"Yes. I would feel unwell when I took previous medications. The doctor asked me about side effects. I told the doctor after I took the medication I would feel pain here, so I wouldn't dare to eat the medications." IDI002_M_81-85_Chinese*
Condition Related Factors	Lack of symptoms	*"For blood pressure, I usually don't take the medications unless my blood pressure is high." IDI014_M_71-75_Chinese*

	Effects on functional status or mental state	*"It is helpful for your knowledge but sometimes it makes you worried when you look at the medicine, you know? It affects you in that way, you know? 'Am I killing myself by taking the medicine, or..?" IDI011_F_61-65_Indian*
